# A "Model Scheme" in Gloucestershire

**Published:** 1920-05-29

**Authors:** 


### THE RED CROSS IN THE SHIRES.

### A "Model Scheme" in Gloucestershire.

We note with pleasure that county organisation, proposed in the statement issued with the appeal from the Joint Council of the British Red Cross and the Order of St. John, for the benefit of the civil hospitals, is already getting to work. One instance is typical. Within the past fortnight Sir Francis Colchester-Wemyss, late Director of the Red Cross organisation in Gloucestershire, has called a meeting in the Shire Hall, Gloucester, to discuss the peace work to be carried out by the county branch of the Joint Council. The meeting concerned itself chiefly with the plan to assist the voluntary hospitals in the area. Of the new preliminary committee Sir Francis is chairman, Miss Allen honorary secretary, while the Rev. R. H. M. Bouth is the county director. The new committee includes Mr. Hicks Beach (chairman of the Extension of Public Health Services Committee); Miss Milford, to represent the Health Visitors; Mr. Stamford Hutton (chairman of the Disablement Committee); Dr. Middleton Martin (County Medical Officer of Health; and Sir Arthur Austin (chairman of the Territorial Force Association). To this nucleus members of other bodies, and presumably the representatives of the voluntary hospitals, will be shortly added. On the military side of the work of the new committees, of which this one serves as a promising type, we need not here dwell. Dr. Middleton Martin is advising what use may, perhaps, be made in school clinics and so forth, of volunteer workers, whom the Ministry of Health seems to favour. Ambulances to serve districts of ten-mile radius apiece, it is planned, will be placed throughout the county. The cost of maintaining each ambulance is not expected to be more than £60 a year.

### To Assist Civil Hospitals.

Sir Francis Colchester-Wemyss, in his opening speech, declared that the chief work of the new committee will be to carry out the scheme to assist civil hospitals. The chief difficulties of the latter, he said, were a shortage of money and a scarcity of trained nurses, especially the latter. It had been proposed, but not decided, that hospitals should be helped from some "outside source" to pay additional salaries, which, perhaps, were still unduly low. If the salaries paid by the hospitals could be supplemented by some external means, he thought that trained nurses would be more easily obtainable.

In regard to finance, he referred to the survey, made by Sir Napier Burnett. In round numbers there were about 800 civil hospitals, with 45,000 beds. Of these 469 had been surveyed, and particulars of a further hundred had been received. The figures, which he thus prefaced, would refer roughly to 67 per cent. of the whole number. Of the hospitals reviewed, the excess of expenditure was about 15 per cent., the figures being: Income, £2,500,000; expenditure, £2,900,000. It was interesting to observe that, including those sent to the Hospital Saturday Funds and those subscribed direct to institutions, the extent of workmen's contributions was 15 per cent., not less, nor more. Of the rest of their income, 44 per cent. came from ordinary subscriptions, 19 per cent. were contributions from public services, 14½ per cent. from investments, and 7 per cent. from patients.

These figures, we may note in passing, tell their own tale, and reveal where the strength of voluntary support mainly resides at present.

### Depreciation of Investment.

In regard to the depreciation of investments, the speaker continued, hospitals had been badly hit, especially since securities had to be sold, and at a loss, to meet current expenditure. In the case of nine hospitals, the depreciation of the value of stock was as much as one-fifth of the total value of their holdings. Everyone knew how the cost of maintenance had increased. He attributed the reduced income to diminished subscriptions, the depreciation aforesaid, and to the withdrawal of military patients. The effect of the latter was that beds lately occupied by paying patients were now occupied by non-paying ones. Even if the grants for military patients did not cover their cost per day, the successors of these patients did not contribute even that much.

### Co-operative Buying.

Apart from the funds received from the appeal of Queen Alexandra, which the Joint Council would administer on the lines of King Edward's Hospital Fund for London, and insist upon co-ordination between hospitals in the county areas, the council would try to start a large scheme of co-operative buying. Drugs, dressings, and perhaps food, their experience during the war had taught them, could thus be bought economically. It might be possible not only to make purchases wholesale, but also to purchase certain goods in the country of their origin, and thus to reduce not only the retailers', but even the wholesalers' profits. Such a scheme if effective, might supplement the financial value of the grants in money, by a saving of 10-15 per cent. in purchase prices. Thus the grants in cash and the saving in expense should make all the difference between solvency and insolvency. At the same time co-ordination would produce invaluable, because co-ordinated, statistics, and make inter-hospital co-operation a living force.

In regard to the scheme for the co-ordination of the health services, Gloucestershire's plan was early in the field, and would be a model to other counties. It was in this work especially that the new committee would be useful. He hoped that the voluntary aid women, who had been working in the hospitals during the war, might act as relief nurses at holiday times in those civil hospitals in the county which needed their assistance.

